# A novel variant in *GLIS3* is associated with osteoarthritis

**DOI:** 10.1136/annrheumdis-2017-211848

**Published:** 2018-02-07

**Authors:** Elisabetta Casalone, Ioanna Tachmazidou, Eleni Zengini, Konstantinos Hatzikotoulas, Sophie Hackinger, Daniel Suveges, Julia Steinberg, Nigel William Rayner, arcOGEN Consortium, Jeremy Mark Wilkinson, Kalliope Panoutsopoulou, Eleftheria Zeggini

**Affiliations:** 1 Department of Medical Sciences, University of Turin, Turin, Italy; 2 Italian Institute for Genomic Medicine, IIGM, Turin, Italy; 3 Human Genetics, Wellcome Trust Sanger Institute, Hinxton, UK; 4 Department of Oncology and Metabolism, University of Sheffield, Sheffield, UK; 5 5th Psychiatric Department, Dromokaiteio Psychiatric Hospital of Athens, Athens, Greece; 6 Wellcome Trust Centre for Human Genetics, Oxford, UK; 7 Oxford Centre for Diabetes, Endocrinology and Metabolism, University of Oxford, Oxford, UK

**Keywords:** osteoarthritis, genome-wide association study, functional genomics, single nucleotide polymorphism

## Abstract

**Objectives:**

Osteoarthritis (OA) is a complex disease, but its genetic aetiology remains poorly characterised. To identify novel susceptibility loci for OA, we carried out a genome-wide association study (GWAS) in individuals from the largest UK-based OA collections to date.

**Methods:**

We carried out a discovery GWAS in 5414 OA individuals with knee and/or hip total joint replacement (TJR) and 9939 population-based controls. We followed-up prioritised variants in OA subjects from the interim release of the UK Biobank resource (up to 12 658 cases and 50 898 controls) and our lead finding in operated OA subjects from the full release of UK Biobank (17 894 cases and 89 470 controls). We investigated its functional implications in methylation, gene expression and proteomics data in primary chondrocytes from 12 pairs of intact and degraded cartilage samples from patients undergoing TJR.

**Results:**

We detect a genome-wide significant association at rs10116772 with TJR (P=3.7×10^−8^; for allele A: OR (95% CI) 0.97 (0.96 to 0.98)), an intronic variant in *GLIS3*, which is expressed in cartilage. Variants in strong correlation with rs10116772 have been associated with elevated plasma glucose levels and diabetes.

**Conclusions:**

We identify a novel susceptibility locus for OA that has been previously implicated in diabetes and glycaemic traits.

## Introduction

Despite the considerable heritability and prevalence of osteoarthritis (OA), there has been limited success in unravelling its genetic aetiology. Genome-wide association studies (GWAS) have established 19 common variants associated with OA reaching or approaching genome-wide significance.^1 2^ Six common variants have been associated with minimal joint space width with two of them also known to be associated with the dichotomous phenotypic definition of OA.^3^ The established, common knee and/or hip OA variants confer small effect sizes and account for just 11% of the disease heritability.^1^ Recently, two rare variations with large effect sizes in hip OA were identified by whole genome sequencing and imputation in a large-scale GWAS of Icelanders: one private to this population and a rare indel that is present in cosmopolitan populations.^4^ It is anticipated that larger sample sizes, harmonised phenotypes and interrogation of rare variation will enable the identification of further OA susceptibility loci.

Here we carried out a GWAS in 5414 OA cases and 9939 controls from the UK genotyped on the Illumina HumanCoreExome Beadchip. Our aim was to interrogate variants from the full minor allele frequency (MAF) spectrum including potentially functional, rare coding variants with modest to large effects sizes in OA. We sought replication of our top signals in individuals with self-reported OA and in individuals with operation due to OA from the UK Biobank resource, in order to improve power by increasing sample size and by harmonising phenotype definition, respectively.

## Methods

Full details of all methods are given in the online supplementary material 1, and the study design is summarised in online supplementary figure S1.

10.1136/annrheumdis-2017-211848.supp1 Supplementary file 1

10.1136/annrheumdis-2017-211848.supp2 Supplementary file 2

Briefly, we genotyped 6214 cases with hip and/or knee OA from the arcOGEN study^5^ on three versions of the Illumina HumanCoreExome Beadchip: 12-v1.0, 12-v1.1 and 24-v1.0 (Ilumina, San Diego, California, USA). As controls, we used individuals from the UK Household Longitudinal Study^6^ genotyped on the same chip (12-v1.0). Genotypes were called with GenCall, and standard GWAS quality control (QC) was conducted for each chip version separately at the sample and variant levels. We employed additional QC approaches to guard against chip effects (online  supplementary material, figures S2 and S3). Following the data merge at the intersection of clean samples and variants, we included 5414 cases that were ascertained by total joint replacement (TJR) surgery, 9939 controls and 270 934 variants in downstream analyses. We performed three association analyses for knee and/or hip TJR and stratified by joint. We prioritised variants with P<10^−4^ for replication in OA case/control sets from the interim release of UK Biobank.^7^ Replication sets comprised either individuals with self-reported OA versus non-OA controls (12 658 cases versus 50 898 controls) or individuals with hip and/or knee operation hospitalisation codes versus non-OA controls (up to 4015 cases versus 16 060 controls) (online supplementary material, tables S1 and S2). We followed up our lead finding in 17 894 operated cases versus 89 470 non-OA controls from the full release of UK Biobank.^8^

We performed fine mapping and bioinformatics queries in the region of association at the chr9 locus following imputation. Finally, we followed up the signal at rs10116772 using functional genomics data (DNA methylation, gene expression and protein abundance) in primary chondrocytes from matched intact and degraded cartilage from 12 patients with OA undergoing total knee replacement.^9^ The data were analysed for significant differences between intact and degraded cartilage on the three -omics levels.^9^

## Results

We performed association analyses for three OA strata in the discovery study versus all 9939 controls (online supplementary table S3). Quantile–quantile plots and Manhattan plots are shown in online supplementary figures S4 and S5. A total of 94 variants (representing 68 independent signals) with P<10^−4^ were prioritised for replication. Fifty-eight of these variants were not present in the previous arcOGEN GWAS^5^ due to limited overlap of single nucleotide polymorphism (SNP) content between platforms (online supplementary material, tables S4 and S5). Seventy-six of the prioritised variants were present in the interim UK Biobank dataset post-QC and underwent meta-analyses (online supplementary table S6).

10.1136/annrheumdis-2017-211848.supp7 Supplementary file 7

10.1136/annrheumdis-2017-211848.supp12 Supplementary file 12

We empirically evaluated replication power in three main OA phenotype classifications in the UK Biobank study: self-reported OA (12 658 cases, 50 898 controls), hospital diagnosed OA (10 083 cases, 40 425 controls) and a subset of hospital-diagnosed OA subjects with operation codes (4015 cases, 16 060 controls) (online supplementary material, tables S1 and S2). For the established OA loci, we find that the self-reported dataset is the only single study in Europeans that identifies the well-established signal for OA at the growth/differentiation factor 3 gene (*GDF5*) with genome-wide significance. We also find that the much smaller, operation OA set is more powerful to detect variants associated with the more homogeneous, severe endpoint of TJR and/or with joint specificity (online supplementary material and table S7). The evaluation of the accuracy of self-reported OA status when compared with hospitalisation records shows modest sensitivity and high specificity, in line with results from other diseases studied in UK Biobank.^10^ These assessments underpinned our decision to include replication from the self-reported and from the operation OA phenotype definitions for OA at any site and stratified by site.

rs10116772 (frequency of 0.40) reached genome-wide significance with P=3.2×10^−8^ and OR (95% CI) 0.97 (0.96 to 0.98) following the meta-analysis of knee and/or hip OA TJR with the self-reported OA case–control dataset (online supplementary table S6). The direction of effect is concordant in the replication operation set versus controls, but the power decreases (meta-analysis P=1.4×10^−6^), commensurate with the significant decrease in sample size compared with the self-reported OA set (68% less samples) (online supplementary table S6). As a sensitivity analysis, we followed up this locus in the intermediate-sized dataset of hospital diagnosed OA versus controls and find concordant effect size (OR (95% CI) 0.97 (0.94 to 1)) and 2.6-fold higher power (P=0.07) than in the operation subset (P=0.18). Motivated by this finding, we followed up the association of rs10116772 in operated OA cases from the full release of the UK Biobank data, and we replicate it with genome-wide significance (P=3.7×10^−8^, OR (95% CI) 0.97 (0.96 to 0.98) for the same phenotype as the discovery study (table 1).

**Table 1:** Summary statistics of rs10116772 in the discovery and replication studies

Study	Stratum	n (case/control)	EA	EAF	P value	OR (95% CI)
Discovery	Knee and/or hip OA TJR versus controls	5410/9936	A	0.40	3.29×10^–6^	0.97 (0.96 to 0.98)
Replication	Knee and/or hip OA operation versus non-OA controls	17 894/89 470	A	0.40	2.32×10^–3^	0.96 (0.94 to 0.99)
Meta-analysis		23 304/99 406	A	0.40	3.71×10^–8^	0.97 (0.96 to 0.98)

EA, effect allele; EAF, effect allele frequency; TJR, total joint replacement.

The association strength of rs10116772 was not attenuated following adjustment for height and body mass index (BMI) in our cohort (online supplementary material and online supplementary table S8). Furthermore, rs10116772 was not significantly associated with any of these traits in large-scale meta-analyses from Genetic Investigation of ANthropometric Traits (GIANT) and from the UK10K Consortia (online supplementary material and table S9).

10.1136/annrheumdis-2017-211848.supp14 Supplementary file 14

**Figure 1:**
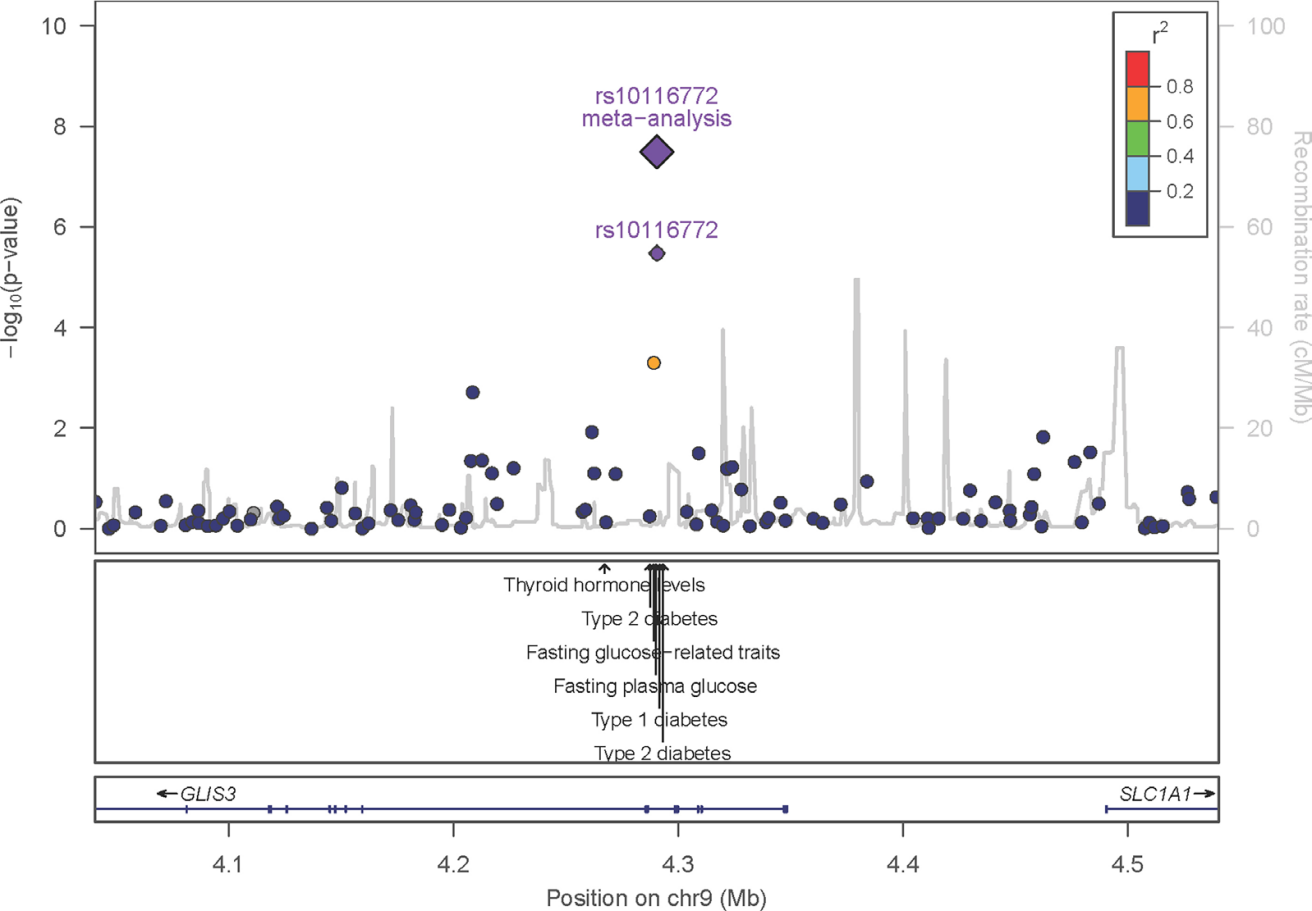
Association of variants at *GLIS3* with osteoarthritis. The x-axis shows the genomic interval, the left y-axis shows the statistical significance of association as negative log10 of P values. rs10116772 was used as the reference SNP and its P value in the discovery set, and postreplication is denoted by the purple circle and diamond, respectively. The linkage disequilibrium is presented as pairwise r^2^ between the reference SNP and the other SNPs in the region with colours according to different bins (0–0.2: dark blue; 0.2–0.4: light blue; 0.4–0.6: green; 0.6–0.8: orange; 0.8–1.0: red). The location of variants associated with other traits as reported in the NHGRI-EBI GWAS catalogue (P<10^−5^) is shown by the arrows. SNP, single nucleotide polymorphism.

The regional association plot shows a narrow region of association in the GLIS Family Zinc Finger 3 (*GLIS3*) gene (figure 1). Imputation of this locus, fine mapping and bioinformatics follow-up do not confidently predict the causal variant (online supplementary material, figure S6, tables S10 and S11). rs10116772 is in near-perfect LD (r^2^>0.99) with a genome-wide significant variant for fasting plasma glucose levels (rs4237150) and in moderate LD (r^2^>0.6) with variants that are associated with type 1 diabetes, type 2 diabetes and glycaemic traits (online supplementary table S12).

10.1136/annrheumdis-2017-211848.supp19 Supplementary file 19

**Figure 2:**
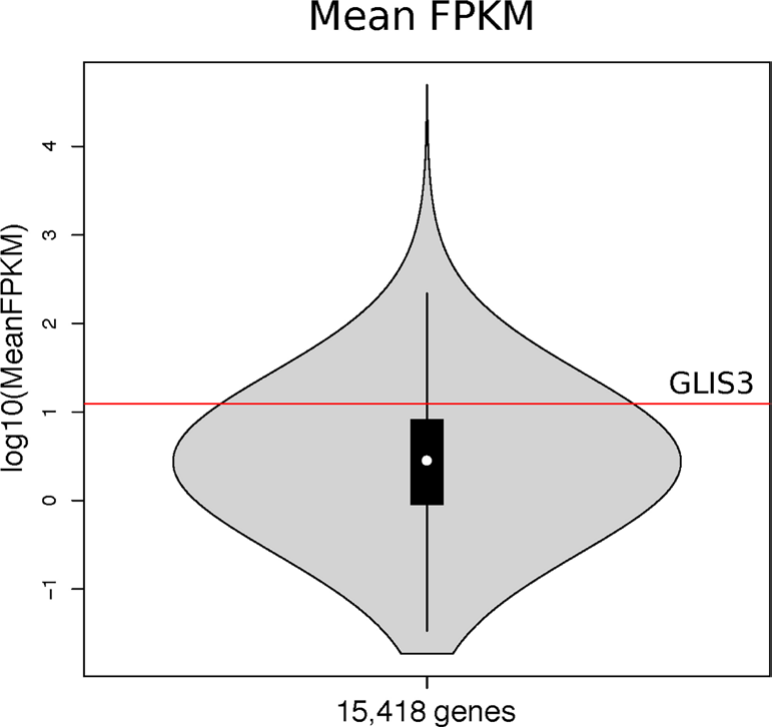
Violin plot of the expression levels of *GLIS3* based on intact cartilage from 12 individuals undergoing knee replacement surgery for primary osteoarthritis. The mean expression level of *GLIS3* (log10(MeanFPKM)) is shown by the red line. The inner black box shows the interquartile range (IQR, 25%–75% percentiles), the white dot is the median and the whiskers are the furthest point with distance not more than 1.5 times the IQR away from the median. FPKM, fragments per kilobase of transcript per million mapped reads.

*GLIS3* is expressed in cartilage at 12.5 mean fragments per kilobase of transcript per million mapped reads (FPKM), with SE 1.1 and range 7.6–20.8, calculated based on 12 intact cartilage samples (figure 2). We do not detect differences in the expression, methylation and proteomics analyses between intact and degraded cartilage (online supplementary material).

## Discussion

We carried out a GWAS for OA TJR cases and controls genotyped on the CoreExome chip allowing us to interrogate variants from the full MAF spectrum. Our study is well powered (>80%) to detect common variants with small effect sizes at P=5×10^−8^, for example, an OR of 1.12 for a variant with a frequency of 0.45%. For low-frequency (MAF 1%–5%) and rare variants (MAF <1%), the study is well powered to detect variants with modest to large effects sizes (OR >1.2 and 1.45, respectively). Within the power constraints of our study, we do not detect any low frequency or rare variant associations of large effect with OA. This could be due to sample size and the incomplete probing of variation across the lower end of the allele frequency spectrum. For example, rs532464664, the rare indel identified by whole genome sequencing,^4^ is not directly typed in our GWAS or present in the Haplotype Reference Consortium imputation reference panel.

Our empirical evaluation of power in the different phenotype definitions of OA underpins our decision to employ two powerful and complementary replication approaches: replication from the self-reported OA dataset, empowered by its large sample size, and replication with the more homogeneous, severe endpoint of knee and/or hip operation. We identify a replicating genome-wide significant association at rs10116772 with TJR, initially in the meta-analysis with the self-reported disease status of the UK Biobank interim release, which we follow up and establish in operated individuals only after a substantial sample size increase in the full UK Biobank data release. This demonstrates that the self-reported crude OA definition is a powerful tool for establishing genetic associations, which is in line with reports from other diseases studied in UK Biobank.^11–13^

rs10116772 resides in an intron of *GLIS3*, which encodes a nuclear protein functioning as a repressor and activator of transcription.^14^ It is involved in the development of pancreatic beta cells, the thyroid, eye, liver and kidney, and mutations in this gene cause a neonatal diabetes syndrome.^15 16^ *GLIS3* is regulated by the suppressor of fused homologue gene that encodes a negative regulator of hedgehog signalling; modulating hedgehog signalling can attenuate the severity of OA.^17^ We find that *GLIS3* is expressed in cartilage but we do not—and are not well powered to—detect differences in expression between intact and degraded articular cartilage to evaluate if the locus is associated with OA progression.

Within 4 kb and in strong correlation with the index variant (r^2^>0.6), there are a number of previously associated variants with type 1 diabetes, type 2 diabetes and related traits such as fasting plasma glucose. The alleles that lower fasting plasma glucose and increase the risk of type 1 and type 2 diabetes also increase the risk of OA (online supplementary table S12).

There is significant genetic correlation of OA with BMI and with type 2 diabetes genome wide, suggesting shared genetic aetiology.^10^ Mendelian randomisation analyses indicate that obesity, but not type 2 diabetes, has a causal effect on OA.^10^ We have previously found an association at the *FTO* gene shared between obesity, type 2 diabetes and OA exerting its effect on OA and diabetes through increased BMI.^18 19^ This finding presents the first genetic link of OA and diabetes that is not exerted via increased risk of obesity, but the exact mechanism of OA susceptibility warrants further investigation.

10.1136/annrheumdis-2017-211848.supp3 Supplementary file 3

10.1136/annrheumdis-2017-211848.supp4 Supplementary file 4

10.1136/annrheumdis-2017-211848.supp5 Supplementary file 5

10.1136/annrheumdis-2017-211848.supp6 Supplementary file 6

10.1136/annrheumdis-2017-211848.supp8 Supplementary file 8

10.1136/annrheumdis-2017-211848.supp9 Supplementary file 9

10.1136/annrheumdis-2017-211848.supp10 Supplementary file 10

10.1136/annrheumdis-2017-211848.supp11 Supplementary file 11

10.1136/annrheumdis-2017-211848.supp13 Supplementary file 13

10.1136/annrheumdis-2017-211848.supp15 Supplementary file 15

10.1136/annrheumdis-2017-211848.supp16 Supplementary file 16

10.1136/annrheumdis-2017-211848.supp17 Supplementary file 17

10.1136/annrheumdis-2017-211848.supp18 Supplementary file 18
