# Supplementary material for: A novel variant in GLIS3 is associated with osteoarthritis
Source: Ann Rheum Dis. 2018 Feb 7;77(4):620–3. doi: 10.1136/annrheumdis-2017-211848 (PMC5890630; doi:10.1136/annrheumdis-2017-211848)
Supplement: Supplementary file 1 [file annrheumdis-2017-211848supp001.docx]

**Online Supplementary Material**

**arcOGEN Consortium authors and affiliations**

John Loughlin^1^, Nigel Arden^2^, Fraser Birrell^3,4^, Andrew Carr^2^, Panos Deloukas^5,6^, Michael Doherty^7^, Andrew W. McCaskie^8,9^, William E. R. Ollier^10^, Ashok Rai^11^, Stuart H. Ralston^12^, Tim D. Spector^13^, Ana M. Valdes^7^, Gillian A. Wallis^14^, J. Mark Wilkinson^15^, Eleftheria Zeggini^16^.

1. Musculoskeletal Research Group, Institute of Cellular Medicine, Newcastle University, Newcastle-upon-Tyne, NE2 4HH, UK.

2. Botnar Research Centre, University of Oxford, Nuffield Orthopaedic Centre, Oxford, OX3 7LD UK.

3. Musculoskeletal Research Group, Institute of Cellular Medicine, Newcastle University, Newcastle upon-Tyne, NE2 4HH, UK.

4. Northumbria Healthcare NHS Foundation Trust, Wansbeck General Hospital, NE63 9JJ, UK.

5. William Harvey Research Institute, Barts and The London School of Medicine and Dentistry, Queen Mary University, London, EC1M 6BQ, UK.

6. Princess Al-Jawhara Al-Brahim Centre of Excellence in Research of Hereditary Disorders (PACER-HD), King Abdulaziz University Jeddah, 21589, Saudi Arabia.

7. Academic Rheumatology, School of Medicine, University of Nottingham, UK, Nottingham, NG5 1PB, UK.

8. Division of Trauma and Orthopaedic Surgery, Department of Surgery, University of Cambridge Cambridge, CB2 0QQ, UK.

9. Musculoskeletal Research Group, Institute of Cellular Medicine, Newcastle University Newcastle-upon-Tyne, NE2 4HH, UK.

10. Centre for Integrated Genomic Medical Research, University of Manchester, Manchester, M13 9PT, UK.

11. Worcestershire Acute Hospitals NHS Trust, Worcester, UK.

12. Centre for Genomic and Experimental Medicine, Institute of Genetics and Molecular Medicine, University of Edinburgh, Edinburgh, EH4 2XU, UK.

13. Department of Twin Research and Genetic Epidemiology, King's College London, London, SE1 7EH, UK.

14. Wellcome Trust Centre for Cell Matrix Research, University of Manchester, Manchester M13 9PT UK.

15. Department of Oncology and Metabolism, University of Sheffield, Sheffield, UK.

16. Wellcome Trust Sanger Institute, Wellcome Genome Campus, Hinxton, CB10 1HH, UK.

**UKHLS authors and affiliations**

Michaela Benzeval^1^, Jonathan Burton^1^, Nicholas Buck^1^, Annette Jäckle^1^, Meena Kumari^1^, Heather Laurie^1^, Peter Lynn^1^, Stephen Pudney^1^, Birgitta Rabe^1^, Dieter Wolke^2^

1. Institute for Social and Economic Research

2. University of Warwick

**Supplementary Methods**

**Discovery samples**

**Cases**. The osteoarthritis cases (OA) used in the discovery genome-wide association study (GWAS) were collected at nine sites in the United Kingdom (UK) by the arcOGEN (arc Osteoarthritis Genetics) Consortium as previously described^1^. The majority of cases were ascertained on the basis of total joint replacement (TJR) surgery with a minority of cases ascertained by the Kellgren-Lawrence grading (KL>=2)^2^. This study utilised cases ascertained by TJR. The collections were approved by the National Research Ethics Service in the United Kingdom, and all subjects provided written, informed consent prior to inclusion.

**Controls.** As controls we used individuals from the United Kingdom Household Longitudinal Study (UKHLS) also known as Understanding Society (<https://www.understandingsociety.ac.uk>). UKHLS is a longitudinal panel survey of 40.000 UK households (England, Scotland, Wales and Northern Ireland) representative of the UK population^3^. Participants are surveyed annually since 2009 and contribute information relating to their socioeconomic circumstances, attitudes, and behaviours via a computer assisted interview. The study includes phenotypical data for a representative sample of participants for a wide range of social and economic indicators as well as a biological sample collection encompassing biometric, physiological, biochemical, and haematological measurements and self-reported medical history and medication use. The United Kingdom Household Longitudinal Study has been approved by the University of Essex Ethics Committee and informed consent was obtained from every participant.

**Genotyping and quality control (QC)**

A total of 6,214 cases from the arcOGEN study and 10,484 controls from the UKHLS study were genotyped on the Illumina HumanCoreExome Beadchip (Ilumina, San Diego, USA) at the Wellcome Trust Sanger Institute (WTSI). Cases were genotyped in batches in three versions of the Human CoreExome Beadchip: 1318 samples were genotyped in 12-sample arrays version 1.0 (12-v1.0); 4066 samples were genotyped in 12-sample arrays version 1.1 (12-v1.1); and 742 samples were genotyped in 24-sample arrays version v1.0 (24-v1.0). Controls were previously genotyped on version 12-v1.0. Genotype calling was carried out with Illumina’s GenCall algorithm and sample and variant QC was performed in separate for each batch prior to pooling the genotypes into one dataset. Unless otherwise stated all QC was carried out in PLINK^4^. Quality control (QC)at the sample level was carried out using autosomal variants that had more than 90% call rate and excluded samples based on the following criteria: samples with missing data proportion >=3%; samples with discordant sex information between reported and genetically estimated genetic sex from chrX: samples with average genome-wide heterozygosity more than 3 standard deviations (SD) either side of the mean of the distribution, calculated separately for common and rare variants (a minor allele frequency (MAF) of less than 1% was used to classify variants as rare); and ethnic outliers identified by multidimensional scaling analysis after merging independent variants from the study data (i.e. only variants with a linkage-disequilibrium r^2^ <0.2) with genotypes from the 1000 Genomes Project resource^5^. We carried out an IBS/IBD calculation to examine the extent of the relatedness in the data but kept those in and decided to account for this relatedness in the downstream association analysis. Variant QC excluded variants with: >=2% missing data proportion, Hardy-Weinberg Equilibrium exact p-value <10^-4^, cluster separation score < 0.4, non-autosomal variants and variants that were monomorphic in any of the three datasets. In addition we carried out pairwise case/control association analyses of each case dataset versus each other and flagged variants with p<5x10^-8^. We merged the three cases datasets and the controls at the intersection of clean variants following standard sample and variant QC, and performed additional QC for chip effects as discussed below. At this stage the data contained 5,830 cases, 9,939 controls and 287,219 autosomal variants.

**Data merge and additional QC for chip effects**

Although Illumina guaranteed 99% identity between the three versions of the CoreExome chip we guarded for chip effects in the following ways. We genotyped 48 samples from arcOGEN and 48 samples from UKHLS in triplicate in each version of the chip and checked the genotype concordance pairwise for each chip. We additionally preformed principal component analysis (PCA) using the EIGENSOFT smartpca package to calculate 50 principal components (PCs) for each PCA^6^. Plots of successive PCs (PC1 against PC2, PC2 against PC3, and so on) were generated using the R Statistical Programming Language^7^.

We also performed PCA on the full combined arcOGEN and UKHLS samples that passed QC. The PCA of the full arcOGEN and UKHLS data was run twice: once including all variants passing standard QC (N= 287,219), and once excluding regions of extended LD, such as the major histocompatibility complex (N= 277,580). We carried out a test for association pairwise between cases typed on different chips and inspected QQ plots. We checked for differences in missing data proportion by case control status between the cases genotyped in each chip version and the control set using a chi square test of non-random missingness and removed variants with p<10^-4^. Finally we inspected genotype intensity plots of all variants with p<10^-4^ from downstream analysis and removed variants with bad cluster plots from the data. To avoid sampling effects at variants with low frequency counts due to the different sample size of the cases typed in each chip we removed monomorphics and variants with minor allele count (MAC<5) from each chip.

Following all QC steps we extracted individuals with TJR and ended-up with 5,414 cases, 9,939 controls and 270,934 autosomal variants.

**Association analysis and prioritisation**

We carried out case/control association analyses for knee and/or hip OA TJR samples and also stratified by joint. Association analysis of the directly-typed data was carried out with the maximum likelihood ratio test as implemented in GEMMA, which uses a linear mixed model that accounts for relatedness and controls for population structure and other confounding factors in the data^8^. We adjusted for gender and the first four principal components by including them as covariates in the analysis. After inspecting genotype intensity plots for variants with p<10^-4^ from all analyses we removed highly correlated variants (by selecting one variant from a pair of highly correlated variants (with pairwise r^2^>0.8). We prioritised 94 variants for replication representing 68 independent signals (with independence defined as pairwise r^2^<0.2 and distance >500kb). Of these, 76 variants were present in the replication data. BMI and height adjustment analyses were carried out for the index SNP by including them as covariates in the analyses.

**Replication samples**

We followed-up the association of the prioritised variants in data from the interim release of the UK Biobank resource. The UK Biobank resource comprises 500,000 participants aged 40-69 years recruited between 2006 and 2010 in 22 assessment centres throughout the UK^9^. The assessment visit included electronic signed consent; a self-completed touch-screen questionnaire; brief computer-assisted interview; physical and functional measures; and collection of biological samples and genetic data (<http://www.ukbiobank.ac.uk/wp-content/uploads/2011/11/UK-Biobank-Protocol.pdf>). For the OA case/control dataset in the interim release of the UK Biobank we extracted (post-QC) individuals with phenotypes as follows: 12,658 OA cases with self-reported OA at any site and 50,898 non-OA controls; 10,083 hospital diagnosed OA cases (all individuals that have a hospital ICD code for OA any site) and 40,425 non-OA controls; 6,586 hospital diagnosed knee and/or hip cases (all individuals that have a hospital ICD code for OA hip and/or knee) and 26,384 non-OA controls; 2,396 hospital diagnosed hip cases (subset of hospital-diagnosed cases including individuals with ICD hip OA codes only) and 9,593 non-OA controls; 4,462 hospital diagnosed knee cases (subset of hospital-diagnosed cases including individuals with ICD knee OA codes only) and 17,885 non-OA controls; 4,015 operated knee and/or hip OA cases (subset of hospital diagnosed cases that have knee and/or hip operation ICD codes) and 16,060 non-OA controls; 2,282 operated hip OA cases (subset of hospital diagnosed cases that have hip operation ICD codes) and 9,193 non-OA controls; and 2,281 operated knee OA cases (subset of hospital diagnosed cases that have hip operation ICD codes) and 9,194 controls. The inclusion criteria for the cases in each category and the exclusion criteria for the controls are shown in Table S1. For the OA case/control dataset in the full release of UK Biobank we extracted (post-QC) 17,894 operated knee and/or hip OA cases and 71,576 controls; 9,650 operated hip OA cases and 38,600 controls and 9,133 operated knee OA cases and 36,532 controls. Controls are partly shared between datasets (Table S2) because for each case/control dataset four times the number of cases were selected as controls (we selected the older participants, males-females balanced). This ratio maximises power (power gains are incremental beyond this ratio) whilst avoiding case-control imbalance; statistical tests are not robust if the ratio is skewed especially for low-frequency and rare variants.^10^

**Replication samples genotyping, QC and data-analysis**

50,000 samples were genotyped using UKBiLEVE array and the rest with UK BioBank Axiom array (Affymetrix). The UK BioBank Axiom is an update of UKBiLEVE. The two arrays share 95% common content.

All the analyses were carried out in Europeans. Subjects with high heterozygosity, low call rate, related participants, and pregnant women were further excluded from analyses. Variant genotypes were called by Affymetrix, and any variants failed by Affymetrix batch-specific QC thresholds were set to missing in all subjects from that batch. Additional SNP QC steps were carried out by the UK Biobank team, in which SNPs at certain batch/plates were set to missing if their genotype distributions were significantly different from other batches/plates (P-value <10^-12^), or there were significant deviations of genotype frequencies from those expected under Hardy-Weinberg equilibrium (P-value <10^-12^). Imputation was carried out using IMPUTE 2 with the combined reference panel of 1000 Genomes phase 3 and UK10K data. Any variants imputed, with minor allele frequency of <0.001% were filtered. The data was analysed using the maximum likelihood ratio test as implemented in SNPTEST v2 with the ten first principal components included as covariates. Association results from SNPs with imputation quality score <0.4 were discarded. The first genotypic release of samples by UK Biobank includes 152,736 samples and 806,466 directly typed SNPs after QC and 73,355,667 variants following imputation (all carried out centrally). The QC and imputation of the genetic dataset on the full ~500,000 participants (~96 million variants) are described in detail in ^11^. Association analyses for rs10116772 in the full release of UK Biobank were carried out using the maximum likelihood ratio test as implemented in SNPTEST v2 with the ten first principal components included as covariates.

**Meta-analysis**

We meta-analysed all 3 strata from the discovery set with operation or self-reported OA case/control datasets from the UK Biobank using a fixed-effects, inverse variance model implemented in GWAMA. We carried out 2 types of meta-analyses aimed at maximising power by increased sample size and at harmonising phenotype definition respectively: all arcOGEN TJR strata using the UK Biobank self-reported phenotype for OA because of its large sample size (the largest known OA set to date); all arcOGEN TJR strata with UK Biobank operation OA (for OA at both joints and for each joint in separate).

**Imputation, fine mapping and bioinformatics follow-up**

In order to perform fine mapping in the region of association surrounding the index variant rs10116772 we carried out imputation of the chr9 region using the Haplotype Reference Consortium panel (HRC). HRC comprises 64,976 haplotypes at 39,235,157 SNPs constructed using whole genome sequence data from 20 studies of predominantly European ancestry^12^. We first removed variants with MAF<1% and carried out pre-phasing using EAGLE 2 and imputation using the Sanger imputation server following instructions as described in <https://imputation.sanger.ac.uk/>. We analysed the data using the maximum likelihood ratio test implemented in BOLT-LMM, a linear mixed model software that accounts for population structure and relatedness^13^.

For fine mapping we used variants that were in linkage disequilibrium r^2^ >0.2 with rs10116772 and were contained in the imputed region of association (after filtering the HRC summary statistics to exclude variants with imputation information score <0.4 and MAF<1%). 180 variants were used in this analysis contained in the region chr9:4262697–4298955 (build 37). We implemented the Bayesian fine-mapping method CAVIARBF, which uses association summary statistics and correlations among variants to calculate Bayes’ factors and posterior probabilities of each variant being causal^14^. We assumed a single causal variant in the region and calculated 95% credible sets.

We queried Ensembl, the NHGRI GWAS catalogue, GTex and the Mouse Genome Informatics database (MGI) for further bioinformatics insights into the role of the proximal variants and genes at the locus.

**Supplementary Results**

**Additional QC for chip effects**

We observed good pairwise genotype concordance for the 94 samples that were typed in triplicate between the 2 chips and that passed QC. Average genotype and minor allele concordance between non-missing, overlapping genotypes of the 94 individuals typed in triplicate in each chip was 0.99 in all three pairwise comparisons; the lowest genotype concordance for a marker was 0.95, so no markers were excluded from this step.

Samples typed in triplicate on all three chip versions showed no separation in PCA plots suggesting good concordance in genotyping between chips. We did not observe clustering of samples based on chip version in the larger PCA including all arcOGEN and UKHLS samples (Figure S2). Samples showed separation into three distinct groups in the first four PCs when including regions of complex linkage disequilibrium (LD), which are known to introduce structure. As expected, this grouping disappeared when we removed these regions and repeated the PCA analysis (Figure S3).

**Comparison of this study with the previous arcOGEN GWAS**

This study genotyped 5,830 arcOGEN cases; 3,770 of these cases were previously genotyped in the Illumina 610k platform^1^ that has limited overlap with the CoreExome array (110,985 variants shared between the platforms after QC). All controls differ. Therefore 60% of the Qced CoreExome array variants and 76% of the samples are non-overlapping with the previous arcOGEN GWAS.

In contrast to the previous arcOGEN GWAS, which prioritised only common variants (MAF>5%) with p<10^-5^, this study prioritised variants from the full allelic spectrum with p<10^-4^ including low frequency and rare variants with a high prior probability of being functional (Table S5). Other differences between this study and the previous arcOGEN GWAS are: i) Different genotype calling algorithms (GenCall vs Illuminus); ii) Association analyses with the maximum likelihood ratio test as implemented in GEMMA, which uses a linear mixed model that accounts for relatedness and controls for population structure and other confounding factors in the data. We additionally adjusted for gender and the first four principal components by including them as covariates in the analysis. In the previous arcOGEN GWAS this method was not available and no adjustment was carried out for covariates.

Out of the 94 prioritised variants from this study 36 were present in the Illumina 610K platform. A comparison of the strength of association for these loci between the platforms for the stratum from which they were prioritised from is shown in Table S4. We find 3 variants surpassing our prioritisation threshold of p<10^-4^ in the previous arcOGEN GWAS and all 36 variants having concordant effect of direction between studies.

The *GLIS3* index variant, rs10116772, was not directly-typed in the first arcOGEN GWAS. The best proxy, rs10758593 at chr9:4282083 (r^2^=1 with rs10116772) did not surpass the p<10^-5^ threshold of prioritisation in the TJR (N=5,804) vs. controls (N=11,009) analysis (p=1.95x10^-4^).

**Evaluation of power in different phenotype definitions in the replication samples**

We evaluated power to detect genetic associations in three different phenotype classifications of the replication samples using two approaches: sample size calculations for a characteristic complex disease-associated variant and based on the strength of association of the previously established OA loci with knee and/or hip OA. The three phenotype groupings were self-reported (12,658 cases, 50,898 controls), hospital diagnosed (10,083 cases, 40,425 controls) and operation OA (4,015 cases 16,060 controls) (Table S1). The operation OA is a subset of hospital diagnosed OA and the sample-size correlation between all three datasets are shown in Table S2.

For a characteristic complex disease-associated variant with minor allele frequency (MAF) 30% and allelic odds ratio 1.1, the self-reported, hospital-diagnosed, and operation OA analyses have 80%, 56% and 3% power to detect an effect at genome-wide significance (P<5x10^-8^), respectively.

For the established loci analysis we define a statistically significant threshold of p<0.003 after correcting for the number of independent loci tested (n=18) (Table S7). We find that the most powerful association emanates from the self-reported OA set of the UK Biobank which identifies the well-established signal for OA at *GDF5* with genome-wide significance (p=5.1x10^-9^; OR[95%CIs]=0.91[0.89-0.94]). The strength of association in the hospital diagnosed OA set is decreased (p=3.53x10^-7^) although the effect sizes are unchanged OR[95%CIs]=0.91[0.89-0.94]. In the operation set the strength of association decreases even more (p=6x10^-4^) commensurate with the significant decrease in sample size compared to the self-reported OA set (68% less samples). We note that the signal at *GDF5* was first identified with genome-wide significance in a candidate gene study of hip OA in Asian populations^15^ but it took considerable time and effort from large-scale meta-analyses to reach genome-wide significance in Europeans in the knee stratum^16^. Out of the remaining established OA loci we detect two variants with p<0.003 in the hospital diagnosed set: rs10492367 at *KLHDC5;PTHLH* with p=0.001 in hip OA and rs6976 at *GLT8D1* with p=0.0006 in hip OA, however the association of rs6976 is stronger in the hip operation set (p=3.96x10^-5^). In the operation set we additionally detect rs10948172 at *SUPT3H;CDC5L* (p=0.0007 with knee and/or hip OA) and rs9350591 at *FILIP1;SENP6* with hip OA (p=0.0009).

These findings indicate that the self-reported set of UK Biobank is potentially the most powerful dataset to detect variants associated with OA at more than one site. But they also demonstrate that the much smaller, operation OA has power advantages to detect variants exhibiting joint-specificity, and/or associated with the more severe clinical outcome of joint replacement surgery. The increase in power afforded by increased sample size or by more homogeneous phenotype definition varies between associated variants, which is concordant with our previous discovery of associated loci in arcOGEN.^1^ This finding underpins our decision to include replication from the self-reported and from the operation OA phenotype groupings of the UK Biobank, for OA at any site and for OA stratified by site.

**Association with height and BMI**

Because OA has shown genetic overlap with BMI- and height-associated genetic variants^17-20^ we examined the association of rs10116772 with height and BMI in large-scale meta-analyses from GIANT^21,22^ and from the UK10K studies^23^.

We find that in our cohort, adjustment for BMI and height has negligible effect on the association of rs10116772 with OA (Table S8). rs10116772 is not significantly associated with BMI and height in the GIANT and UK10K meta-analysis (p>0.05); we observe a weak association with height in UK10K (p=5.7x10^-3^ in 51,309 individuals, unpublished data) (Table S9).

**Fine mapping, imputation and bioinformatics follow-up**

The probability of at least 1 causal variant in the region is 0.994. The 95% credible set is given in Table S10. Variant rs4237150 at chr9:4290085 had the highest probability of causality (0.219), followed by variant rs6476839 at chr9:4290823 (0.207) and our index variant rs10116772 at chr9:4290541 (0.190).

The functional consequences of the directly-typed and imputed variants in the associated locus (23 associated variants with r^2^>0.6 with the index variant, all intronic) cannot discriminate which is the causal variant and none of these are present in the GTEx data. EIGEN scores (a measure used to assess the functionality of a variant given the available annotations^24^) point to rs6476839 with the highest functionality while rs10116772 ranks second and rs4237150 ranks fifth in this analysis, but the differences in their scores are very small (Table S11).

**Functional genomics**

The data were analysed for significant differences between intact and degraded cartilage on the three -omics levels: differentially methylated probes at 5% FDR, differentially expressed genes at 5% FDR, and differentially abundant proteins. The differentially methylated probes were also summarised into differentially methylated regions.

None of the genes within 500kb of rs10116772 was located within a differentially methylated region, nor differentially expressed on RNA level at 5% FDR, nor differentially abundant on protein level. There are two CpG sites with differential methylation at 5% FDR within 500kb of rs10116772 (chr9:4290541). The probe cg27204993 (chr9:4662937) located in C9orf68, with 16.9% higher methylation in degraded cartilage (4.9% FDR). This probe does not replicate in an additional functional genomics data set from 17 patients with knee OA (unpublished data). The probe cg14325112 (chr9:4115070) located in *GLIS3* has 32% lower methylation in degraded cartilage (3.9% FDR). It is located in a *GLIS3* intron, but also in a promoter flanking region (ENSR00001299286) which is active in osteoblasts. The differential methylation of this probe replicates in in an additional functional genomics data set from 17 patients with knee OA, and in a third data set from 8 patients with hip OA (same direction, p<0.05 in both; unpublished data). However, none of the genes in the region show differential expression at 5% FDR in cartilage.

**References**

1. arcOGEN Consortium; arcOGEN Collaborators; Zeggini E, Panoutsopoulou K, Southam L, Rayner NW, Day-Williams, et al. Identification of new susceptibility loci for osteoarthritis (arcOGEN): a genome-wide association study. *Lancet.* 2012;**380**:815-823.

2. Kellgren JH, Lawrence JS. Radiological assessment of osteo-arthrosis. *Ann Rheum Dis* 1957;**16**:494-502.

3. Lynn, P. Sample design for Understanding Society. *Understanding Society Working Paper Series 2009-01* 2009.

4. Chang CC, Chow CC, Tellier LC, Vattikuti S, Purcell SM, Lee JJ. Second-generation PLINK: rising to the challenge of larger and richer datasets. *Gigascience* 2015;**4:7** eCollection 2015.

5. 1000 Genomes Project Consortium, Auton A, Brooks LD, Durbin RM, Garrison EP, Kang HM, *et al*. A global reference for human genetic variation. *Nature* 2015;**526**:68-74.

6. Patterson N, Price AL, Reich D. Population structure and eigenanalysis. *PLoS Genet* 2006;**2**:e190.

7. The R Project for Statistical Computing. https://www.r-project.org/

8. Zhou X, Stephens M. Genome-wide efficient mixed-model analysis for association studies. *Nat Genet* 2012;**44**:821-4.

9. Sudlow C, Gallacher J, Allen N, Beral V, Burton P, Danesh J, *et al*. UK biobank: an open access resource for identifying the causes of a wide range of complex diseases of middle and old age. *PLoS Med* 2015;**12**:e1001779.

10. Ma C, Blackwell T, Boehnke M, Scott LJ; GoT2D investigators. Recommended joint and meta-analysis strategies for case-control association testing of single low-count variants. *Genet Epidemiol*. 2013;**37**:539-50.

11. Bycroft C, Freeman C, Petkova D, Band G, Elliott LT, Sharp K, *et al*. Genome-wide genetic data on ~500,000 UK Biobank participants. https://www.biorxiv.org/content/early/2017/07/20/166298

12. McCarthy S, Das S, Kretzschmar W, Delaneau O, Wood AR, Teumer A, *et al*. A reference panel of 64,976 haplotypes for genotype imputation. *Nat Genet* 2016;**48**:1279-83.

13. Loh PR, Tucker G, Bulik-Sullivan BK, Vilhjálmsson BJ, Finucane HK, Salem RM, *et al*. Efficient Bayesian mixed-model analysis increases association power in large cohorts. Nat Genet 2015;**47**:284-90.

14. Chen W, Larrabee BR, Ovsyannikova IG, Kennedy RB, Haralambieva IH, Poland GA, *et al*. Fine Mapping Causal Variants with an Approximate Bayesian Method Using Marginal Test Statistics. *Genetics* 2015;**200**:719-36.

15. Miyamoto Y, Mabuchi A, Shi D, Kubo T, Takatori Y, Saito S, *et al*. A functional polymorphism in the 5' UTR of GDF5 is associated with susceptibility to osteoarthritis. *Nat Genet* 2007; **39**:529-33.

16. Valdes AM, Evangelou E, Kerkhof HJ, Tamm A, Doherty SA, Kisand K, *et al*. The GDF5 rs143383 polymorphism is associated with osteoarthritis of the knee with genome-wide statistical significance.

*Ann Rheum Dis* 2011;**70**:873-5.

17. Panoutsopoulou K, Metrustry S, Doherty SA, Laslett LL, Maciewicz RA, Hart DJ, *et al*. The effect of FTO variation on increased osteoarthritis risk is mediated through body mass index: a mendelian randomisation study. *Ann Rheum Dis* 2014;**73**:2082-6.

18. Frayling TM, Timpson NJ, Weedon MN, Zeggini E, Freathy RM, Lindgren CM, *et al*. A common variant in the FTO gene is associated with body mass index and predisposes to childhood and adult obesity. *Science* 2007;**316**:889-94.

19. Miyamoto Y, Mabuchi A, Shi D, Kubo T, Takatori Y, Saito S, Fujioka M, *et al*. A functional polymorphism in the 5' UTR of GDF5 is associated with susceptibility to osteoarthritis. *Nat Genet* 2007;**39**:529-33.

20. Castaño Betancourt MC, Cailotto F, Kerkhof HJ, Cornelis FM, Doherty SA, Hart DJ, Hofman A, *et al*. Genome-wide association and functional studies identify the DOT1L gene to be involved in cartilage thickness and hip osteoarthritis. *Proc Natl Acad Sci U S A* 2012;**109**:8218-23.

21. Wood AR, Esko T, Yang J, Vedantam S, Pers TH, Gustafsson S, *et al*. Defining the role of common variation in the genomic and biological architecture of adult human height. *Nat Genet* 2014;**46**:1173-1186.

22. Locke AE, Kahali B, Berndt SI, Justice AE, Pers TH, Day FR, *et al*. Genetic studies of body mass index yield new insights for obesity biology. Nature 2015;**518**:197-206.

23. Tachmazidou I, Süveges D, Min JL, Ritchie GRS, Steinberg J, Walter K. Whole-Genome Sequencing Coupled to Imputation Discovers Genetic Signals for Anthropometric Traits. *Am J Hum Genet.* 2017;**100**:865-884

24. Ionita-Laza I, McCallum K, Xu B, Buxbaum JD. A spectral approach integrating functional genomic annotations for coding and noncoding variants. *Nat Genet* 2016;**48**:214-20.

25. Cho YS, Chen CH, Hu C, Long J, Zhang W, *et al.* Meta-analysis of genome-wide association studies identifies eight new loci for type 2 diabetes in East Asians. *Nat Genet* 2012;**44**:67-72.

26. Dupuis J, Langenberg C, Prokopenko I, Saxena R, Soranzo N, Jackson AU, *et al.* New genetic loci implicated in fasting glucose homeostasis and their impact on type 2 diabetes risk. *Nat Genet* 2010;**42**:105-16.

27. Hwang JY, Sim X, Wu Y, Liang J, Tabara Y, Hu C, *et al*. Genome-wide association meta-analysis identifies novel variants associated with fasting plasma glucose in East Asians. *Diabetes* 2015;**64**:291-8.

28. Barrett JC, Clayton DG, Concannon P, Akolkar B, Cooper JD, Erlich HA, *et al*. Genome-wide association study and meta-analysis find that over 40 loci affect risk of type 1 diabetes. *Nat Genet* 2009;**41**:703-7.

29. Grant SF, Qu HQ, Bradfield JP, Marchand L, Kim CE, Glessner JT, *et al.* Follow-up analysis of genome-wide association data identifies novel loci for type 1 diabetes. *Diabetes* 2009;**58**:290-5.

30. Li H, Gan W, Lu L, Dong X, Han X, Hu C, *et al*. A genome-wide association study identifies GRK5 and RASGRP1 as type 2 diabetes loci in Chinese Hans. *Diabetes* 2013;**62**:291.
