# Supplementary figures and images for: A novel variant in GLIS3 is associated with osteoarthritis
Source: Ann Rheum Dis. 2018 Feb 7;77(4):620–3. doi: 10.1136/annrheumdis-2017-211848 (PMC5890630; doi:10.1136/annrheumdis-2017-211848)

**Figure S1.** Genome-wide association study design and follow-up.

TJR, total joint replacement

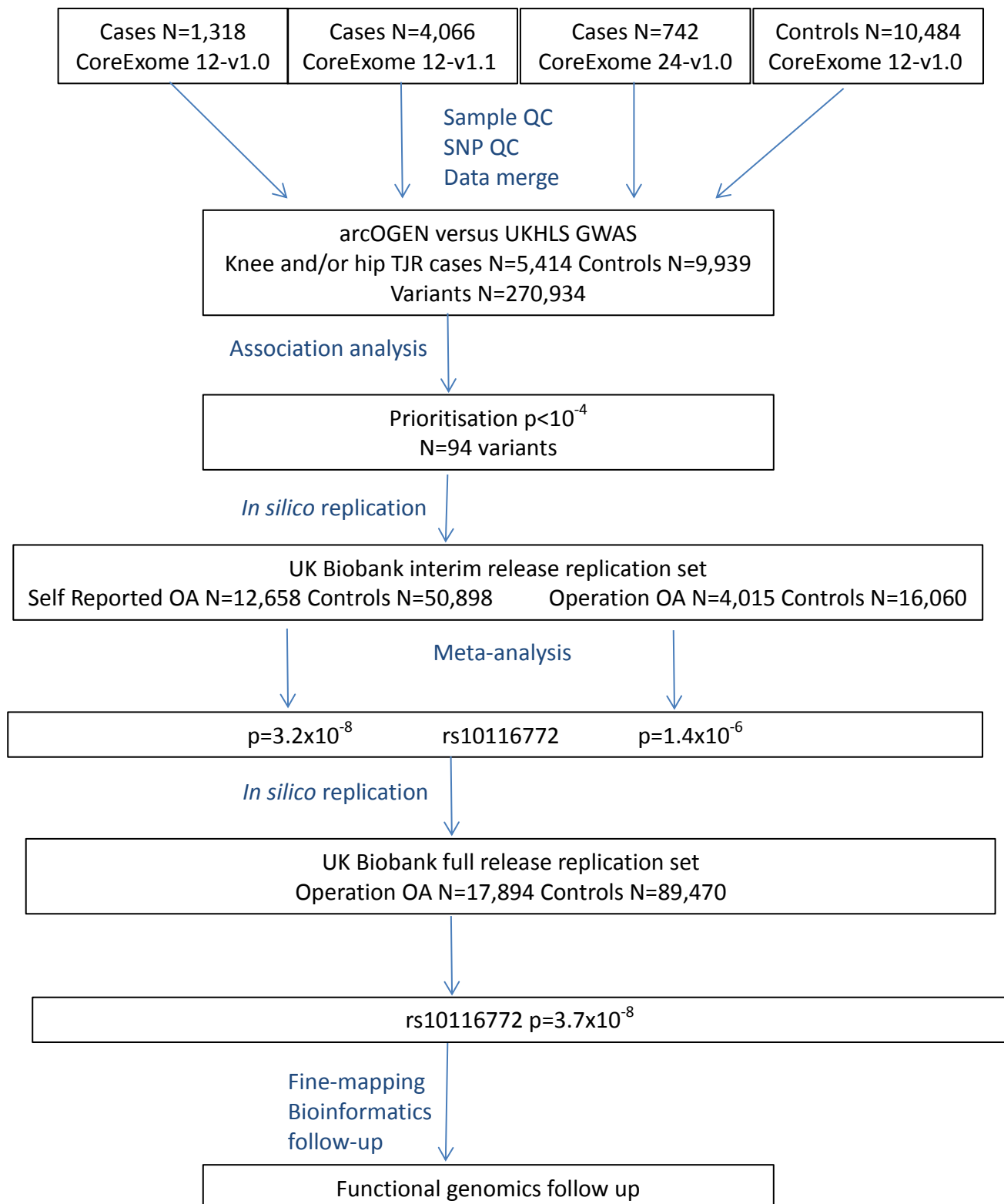

Supplement: Supplementary file 2 [file annrheumdis-2017-211848supp002.pdf]
