# Supplementary material for: A novel variant in GLIS3 is associated with osteoarthritis
Source: Ann Rheum Dis. 2018 Feb 7;77(4):620–3. doi: 10.1136/annrheumdis-2017-211848 (PMC5890630; doi:10.1136/annrheumdis-2017-211848)
Supplement: Supplementary file 3 [file annrheumdis-2017-211848supp003.pdf]

Figure S2. Plots of the first 50 principal components (PCs) from the principal component analysis of arcOGEN and UKHLS samples genotyped in the three different versions of the CoreExome chip. Cases genotyped in 12-sample arrays version 1.0 (12-v1.0) are in red; cases genotyped in 12-sample arrays version 1.1 (12-v1.1) are in green; cases genotyped in 24-sample arrays version v1.0 (24-v1.0) are in blue; controls previously genotyped on version 12-v1.0 are in yellow

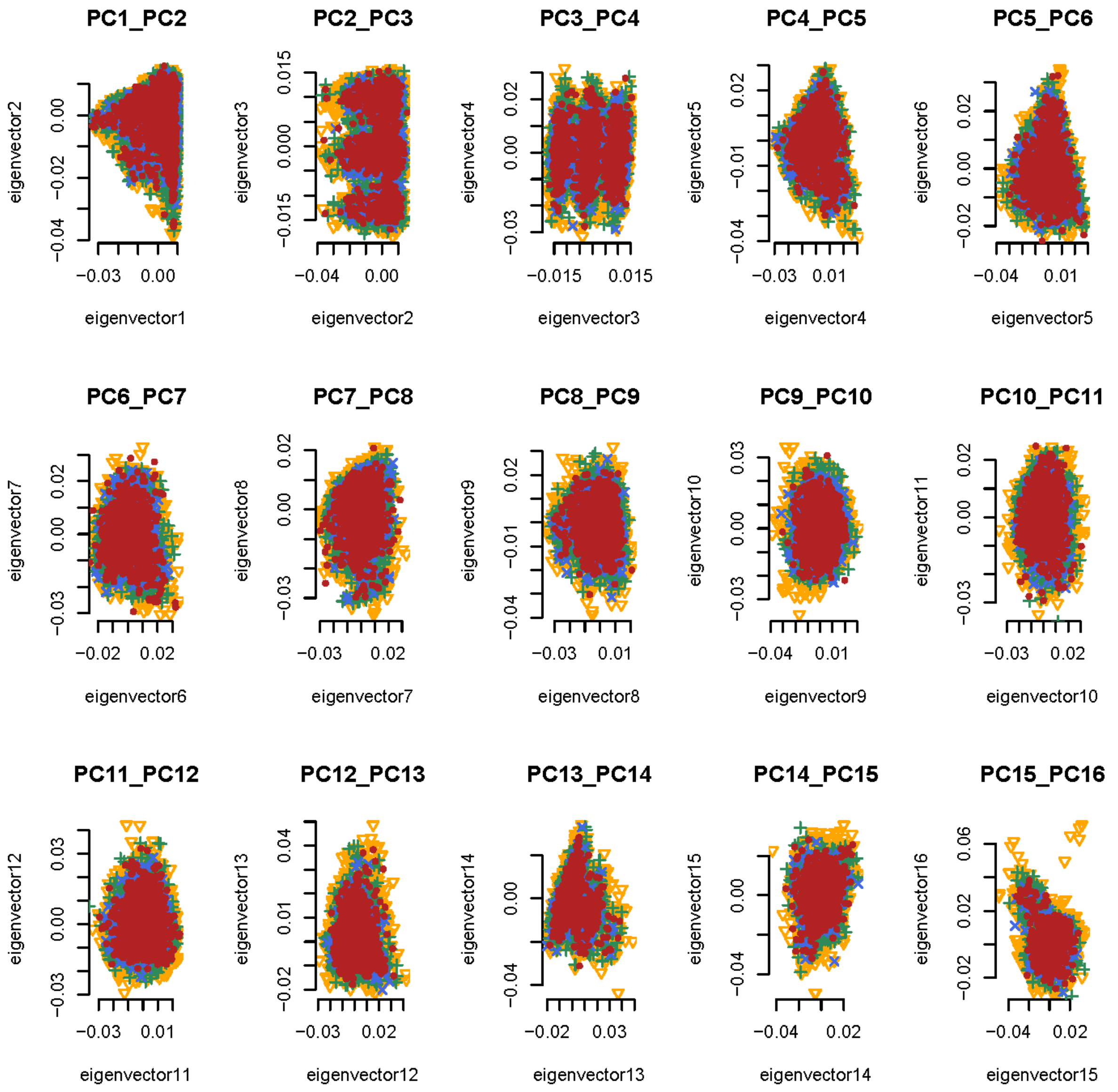

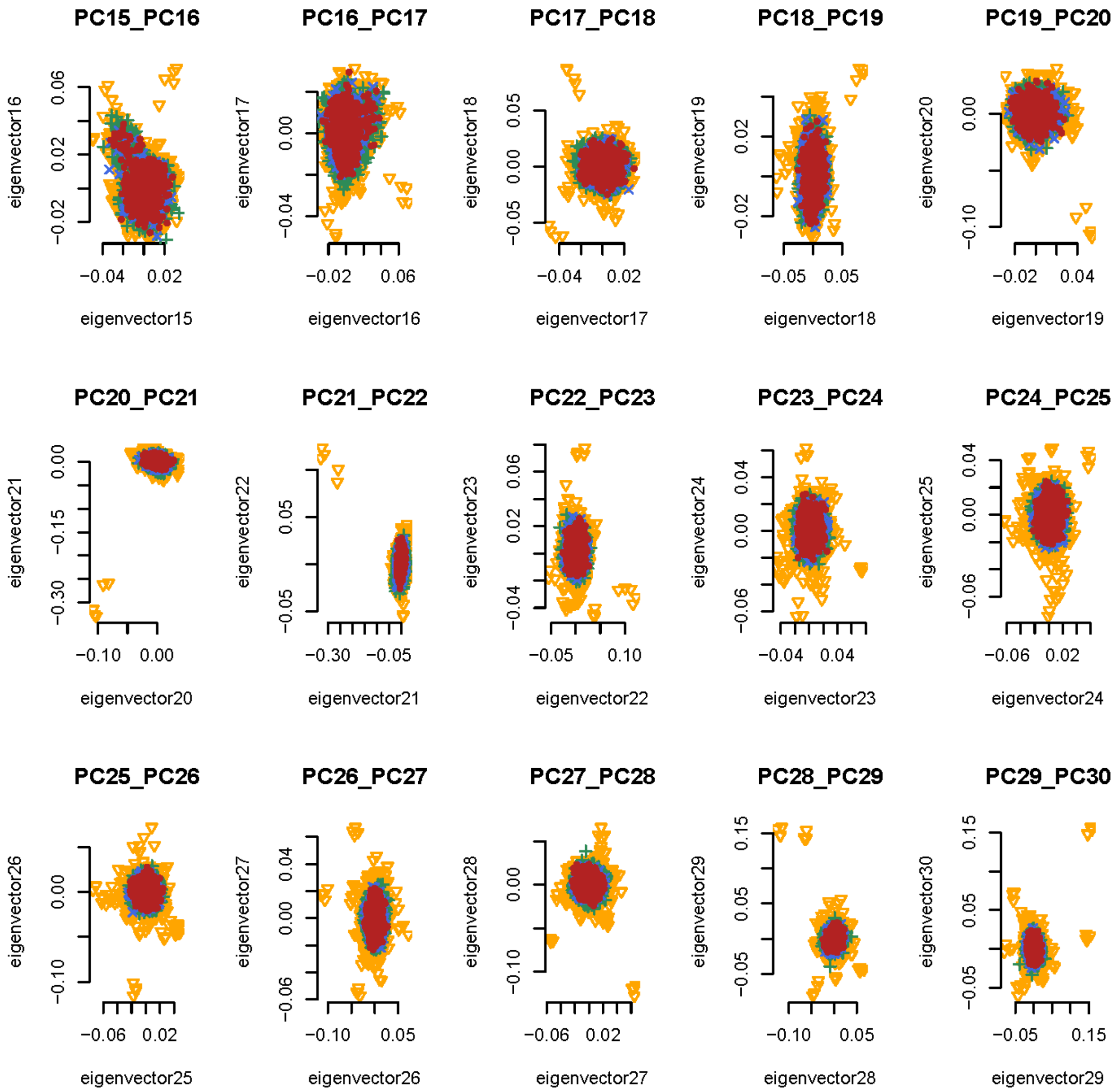
PC15_PC16
eigenvector16
−0.02
0.02
0.06
−0.04
0.02
eigenvector15
PC16_PC17
eigenvector17
−0.04
0.00
−0.02
0.06
eigenvector16
PC17_PC18
eigenvector18
−0.05
0.00
0.05
−0.04
0.02
eigenvector17
PC18_PC19
eigenvector19
−0.02
0.02
−0.05
0.05
eigenvector18
PC19_PC20
eigenvector20
−0.10
0.00
−0.02
0.04
eigenvector19
PC20_PC21
eigenvector21
−0.30
−0.15
0.00
−0.10
0.00
eigenvector20
PC21_PC22
eigenvector22
−0.05
0.05
−0.30
−0.05
eigenvector21
PC22_PC23
eigenvector23
−0.04
0.02
0.06
−0.05
0.10
eigenvector22
PC23_PC24
eigenvector24
−0.06
0.00
0.04
−0.04
0.04
eigenvector23
PC24_PC25
eigenvector25
−0.06
0.00
0.04
−0.06
0.02
eigenvector24
PC25_PC26
eigenvector26
−0.10
0.00
−0.06
0.02
eigenvector25
PC26_PC27
eigenvector27
−0.06
0.00
0.04
−0.10
0.05
eigenvector26
PC27_PC28
eigenvector28
−0.10
0.00
−0.06
0.02
eigenvector27
PC28_PC29
eigenvector29
−0.05
0.05
0.15
−0.10
0.05
eigenvector28
PC29_PC30
eigenvector30
−0.05
0.05
0.15
−0.05
0.15
eigenvector29

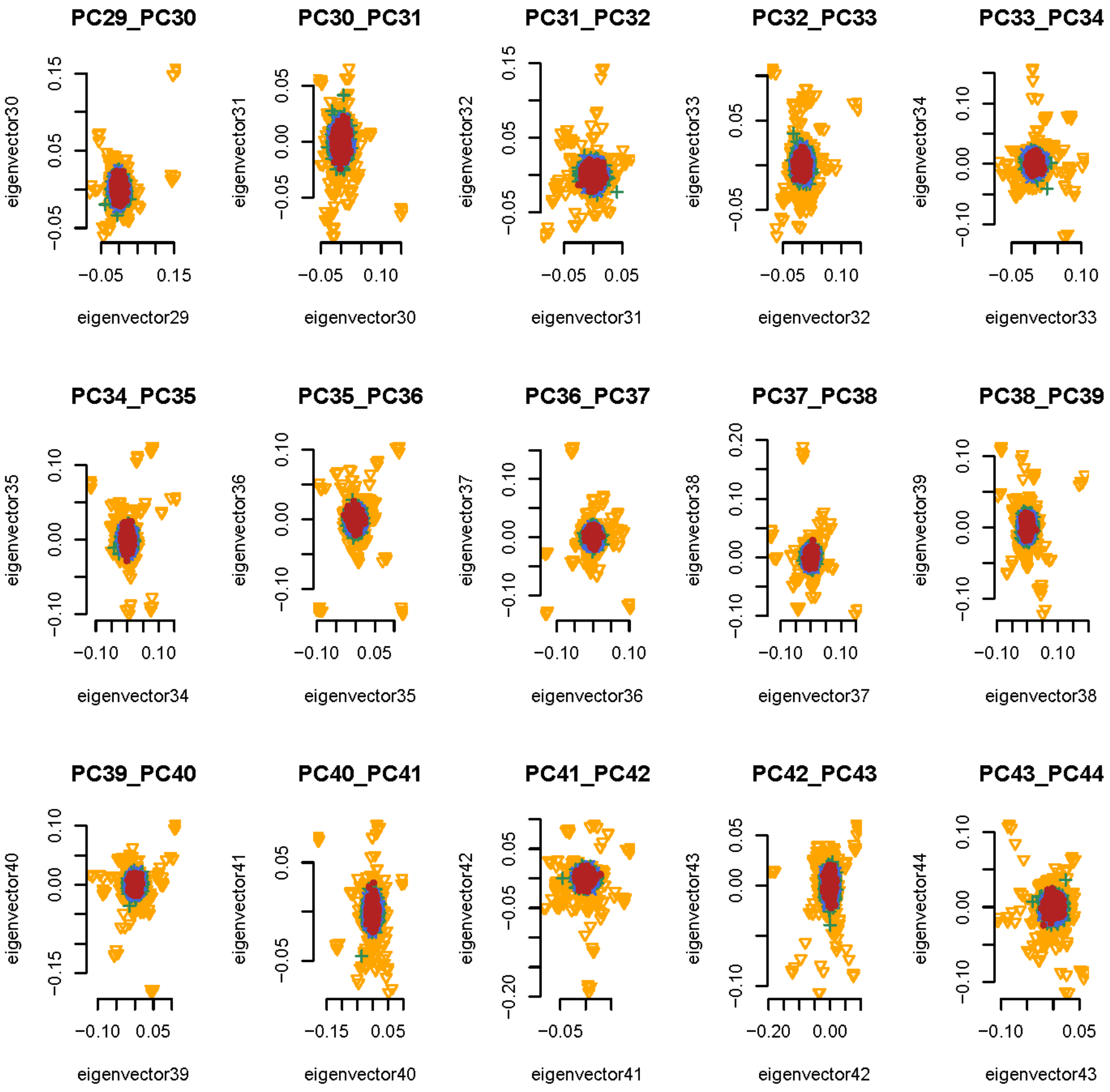
PC29_PC30
eigenvector30
−0.05 0.05 0.15
−0.05 0.15
eigenvector29
PC30_PC31
eigenvector31
−0.05 0.00 0.05
−0.05 0.10
eigenvector30
PC31_PC32
eigenvector32
−0.05 0.05 0.15
−0.05 0.05
eigenvector31
PC32_PC33
eigenvector33
−0.05 0.05
−0.05 0.10
eigenvector32
PC33_PC34
eigenvector34
−0.10 0.00 0.10
−0.05 0.10
eigenvector33
PC34_PC35
eigenvector35
−0.10 0.00 0.10
−0.10 0.10
eigenvector34
PC35_PC36
eigenvector36
−0.10 0.00 0.10
−0.10 0.05
eigenvector35
PC36_PC37
eigenvector37
−0.10 0.00 0.10
−0.10 0.10
eigenvector36
PC37_PC38
eigenvector38
−0.10 0.00 0.10 0.20
−0.10 0.10
eigenvector37
PC38_PC39
eigenvector39
−0.10 0.00 0.10
−0.10 0.10
eigenvector38
PC39_PC40
eigenvector40
−0.15 0.00 0.10
−0.10 0.05
eigenvector39
PC40_PC41
eigenvector41
−0.05 0.05
−0.15 0.05
eigenvector40
PC41_PC42
eigenvector42
−0.20 −0.05 0.05
−0.05
eigenvector41
PC42_PC43
eigenvector43
−0.10 0.00 0.05
−0.20 0.00
eigenvector42
PC43_PC44
eigenvector44
−0.10 0.00 0.10
−0.10 0.05
eigenvector43

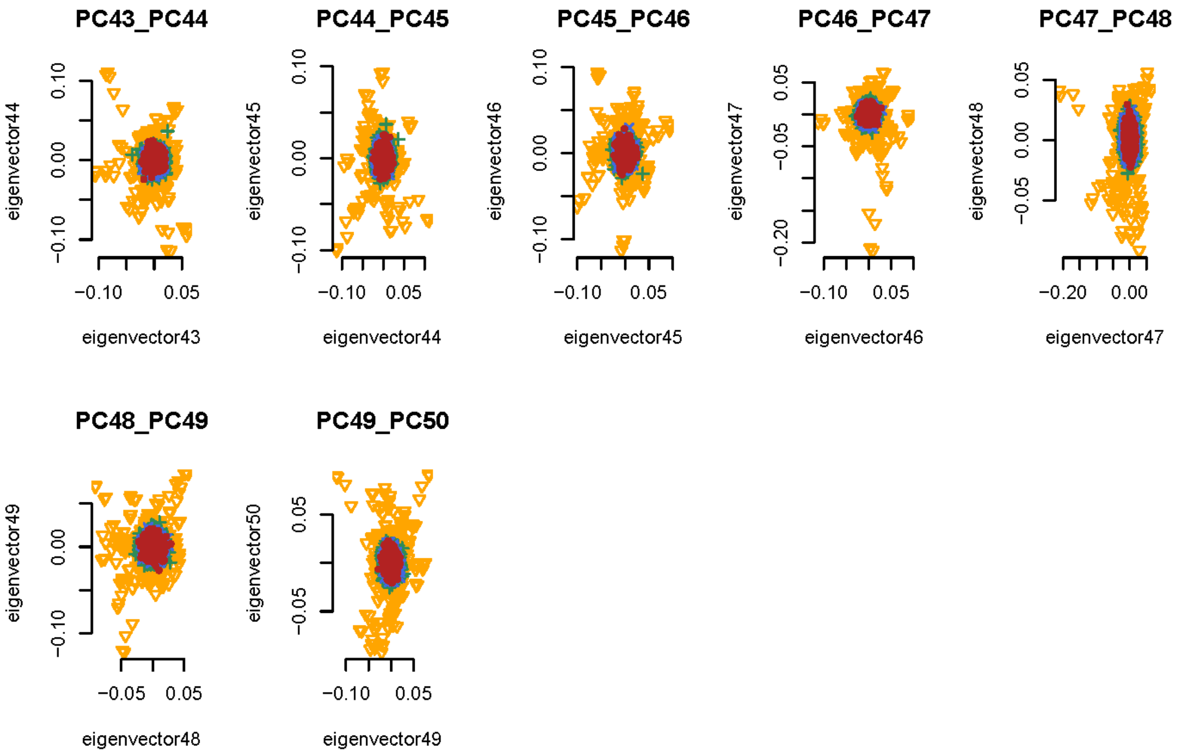
PC43_PC44
eigenvector44
eigenvector43
−0.10
0.00
0.10
−0.10
0.05
PC44_PC45
eigenvector45
eigenvector44
−0.10
0.00
0.10
−0.10
0.05
PC45_PC46
eigenvector46
eigenvector45
−0.10
0.00
0.10
−0.10
0.05
PC46_PC47
eigenvector47
eigenvector46
−0.20
−0.05
0.05
−0.10
0.05
PC47_PC48
eigenvector48
eigenvector47
−0.05
0.00
0.05
−0.20
0.00
PC48_PC49
eigenvector49
eigenvector48
−0.10
0.00
−0.05
0.05
PC49_PC50
eigenvector50
eigenvector49
−0.05
0.05
−0.10
0.05
