# Supplementary material for: A novel variant in GLIS3 is associated with osteoarthritis
Source: Ann Rheum Dis. 2018 Feb 7;77(4):620–3. doi: 10.1136/annrheumdis-2017-211848 (PMC5890630; doi:10.1136/annrheumdis-2017-211848)

Figure S3. Plots of the first 4 principal components (PCs) from the principal component analysis of arcOGEN and UKHLS samples genotyped in the three different versions of the CoreExome chip after excluding complex regions. Cases genotyped in 12-sample arrays version 1.0 (12-v1.0) are in red; cases genotyped in 12-sample arrays version 1.1 (12-v1.1) are in green; cases genotyped in 24-sample arrays version v1.0 (24-v1.0) are in blue; controls previously genotyped on version 12-v1.0 are in yellow.

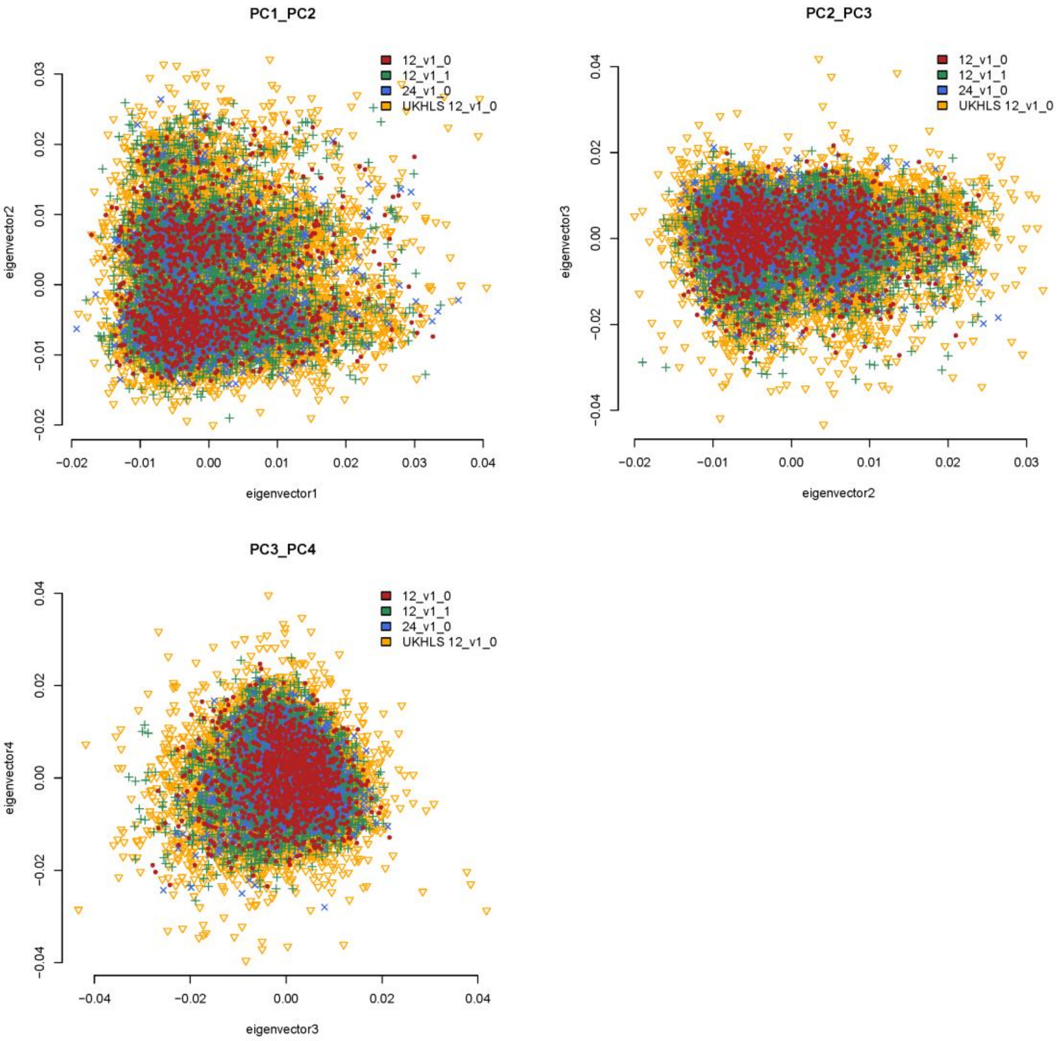

Supplement: Supplementary file 4 [file annrheumdis-2017-211848supp004.pdf]
