# Supplementary material for: A novel variant in GLIS3 is associated with osteoarthritis
Source: Ann Rheum Dis. 2018 Feb 7;77(4):620–3. doi: 10.1136/annrheumdis-2017-211848 (PMC5890630; doi:10.1136/annrheumdis-2017-211848)

Figure S4. Quantile–quantile plots of the OA cases versus controls for the three strata analysed in the discovery GWAS. (a) Knee and/or hip OA, total joint replacement cases versus controls; (b) Hip OA total joint replacement cases versus controls; (c) Knee OA total joint replacement cases versus controls.

(a)

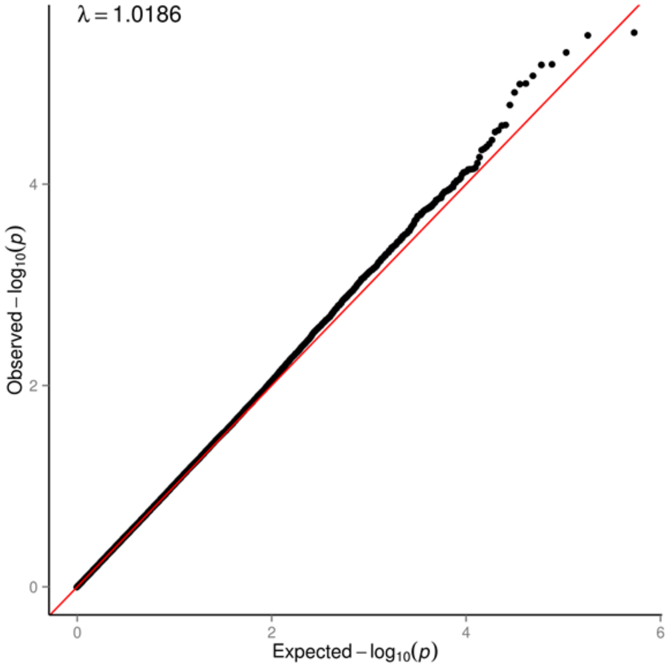

(b)

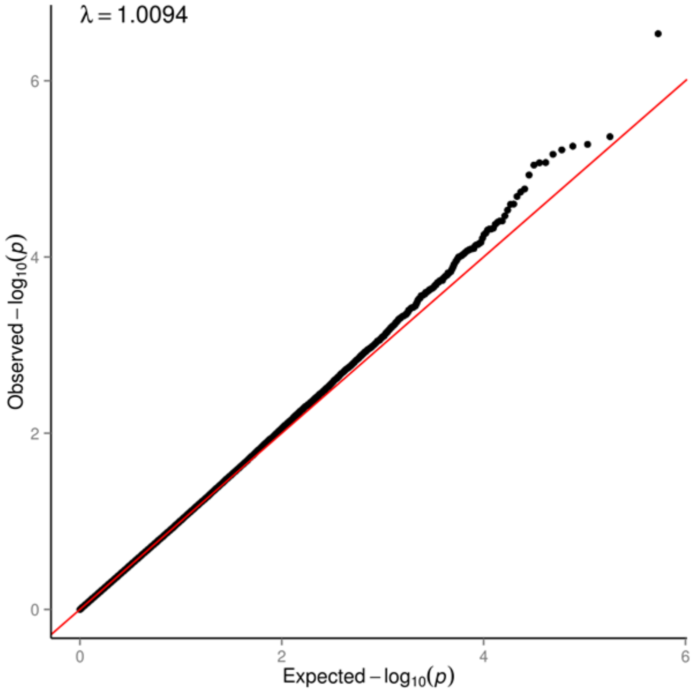

(c)

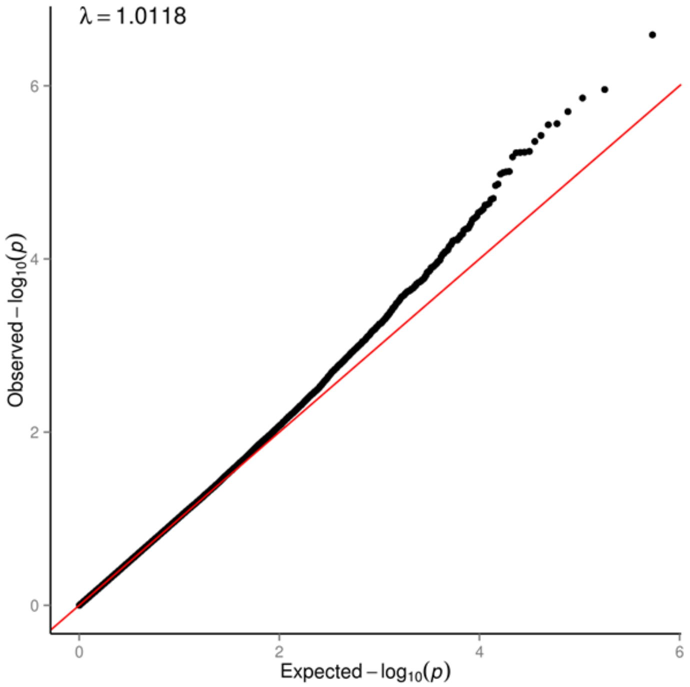

Supplement: Supplementary file 8 [file annrheumdis-2017-211848supp008.pdf]
