# Supplementary material for: A novel variant in GLIS3 is associated with osteoarthritis
Source: Ann Rheum Dis. 2018 Feb 7;77(4):620–3. doi: 10.1136/annrheumdis-2017-211848 (PMC5890630; doi:10.1136/annrheumdis-2017-211848)
Supplement: Supplementary file 9 [file annrheumdis-2017-211848supp009.pdf]

Figure S5. Manhattan plots of the OA cases versus controls association analyses for the three strata analysed in the discovery study. (a) Knee and/or hip OA, total joint replacement cases versus controls; (b) Hip OA total joint replacement cases vs controls; (c) Knee OA total joint replacement cases vs controls.

(a)

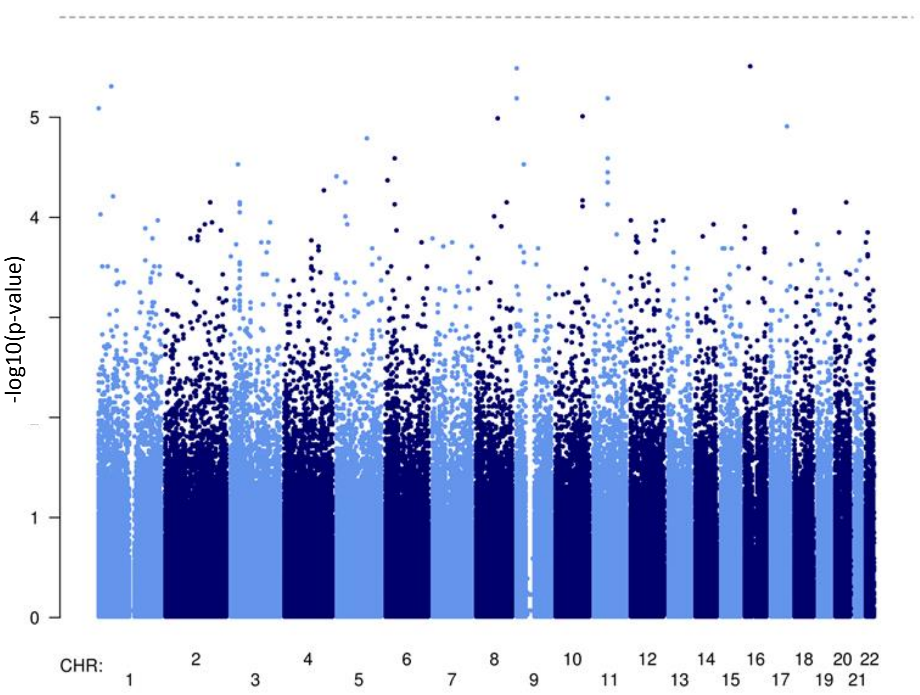

(b)

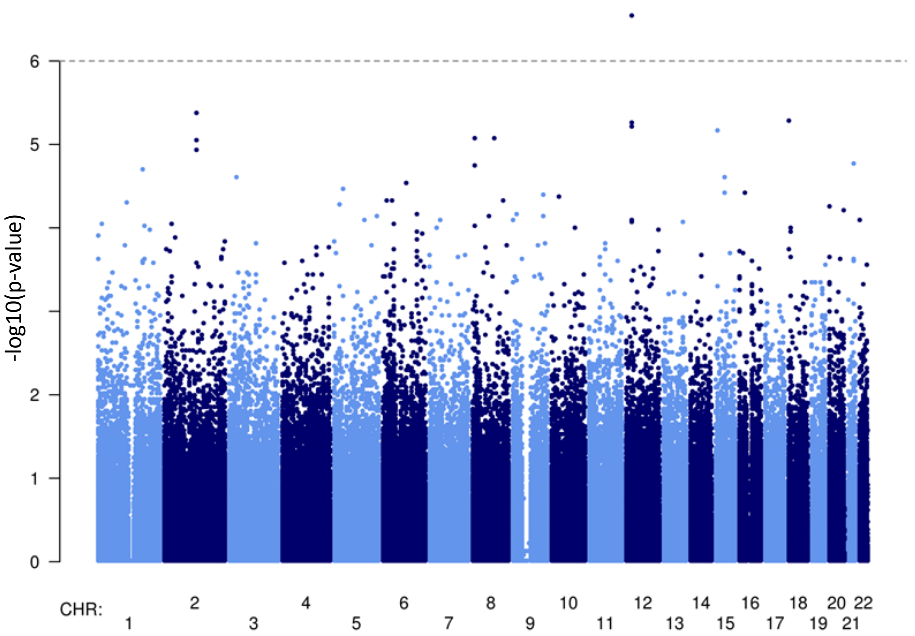

(c)

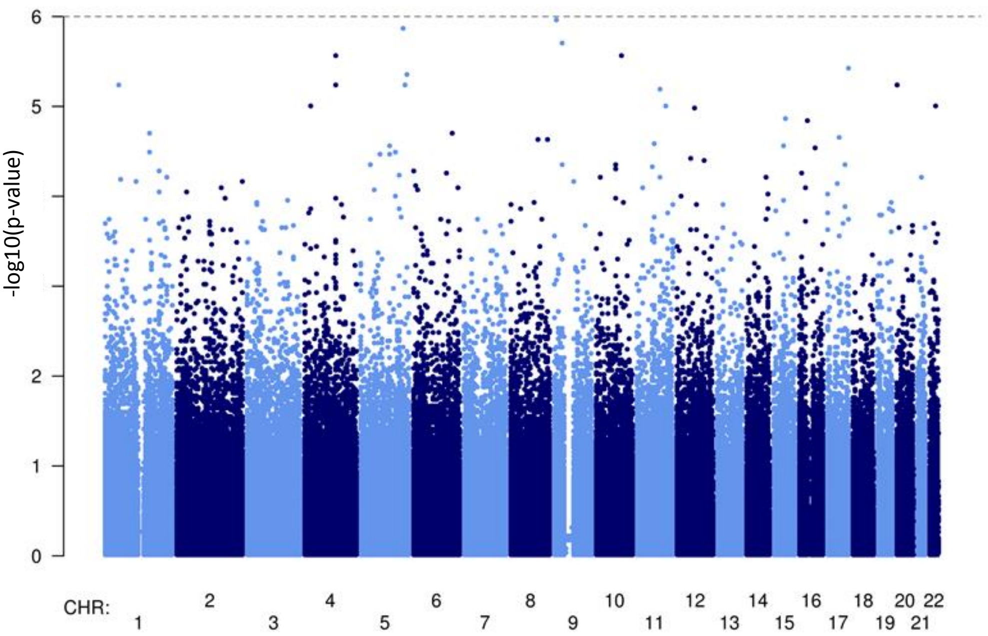

-log10(p-value)
6
5
2
1
0
CHR:
1
2
3
4
5
6
7
8
9
10
11
12
13
14
15
16
17
18
19
20
21
22
