# Supplementary material for: A novel variant in GLIS3 is associated with osteoarthritis
Source: Ann Rheum Dis. 2018 Feb 7;77(4):620–3. doi: 10.1136/annrheumdis-2017-211848 (PMC5890630; doi:10.1136/annrheumdis-2017-211848)
Supplement: Supplementary file 16 [file annrheumdis-2017-211848supp016.pdf]

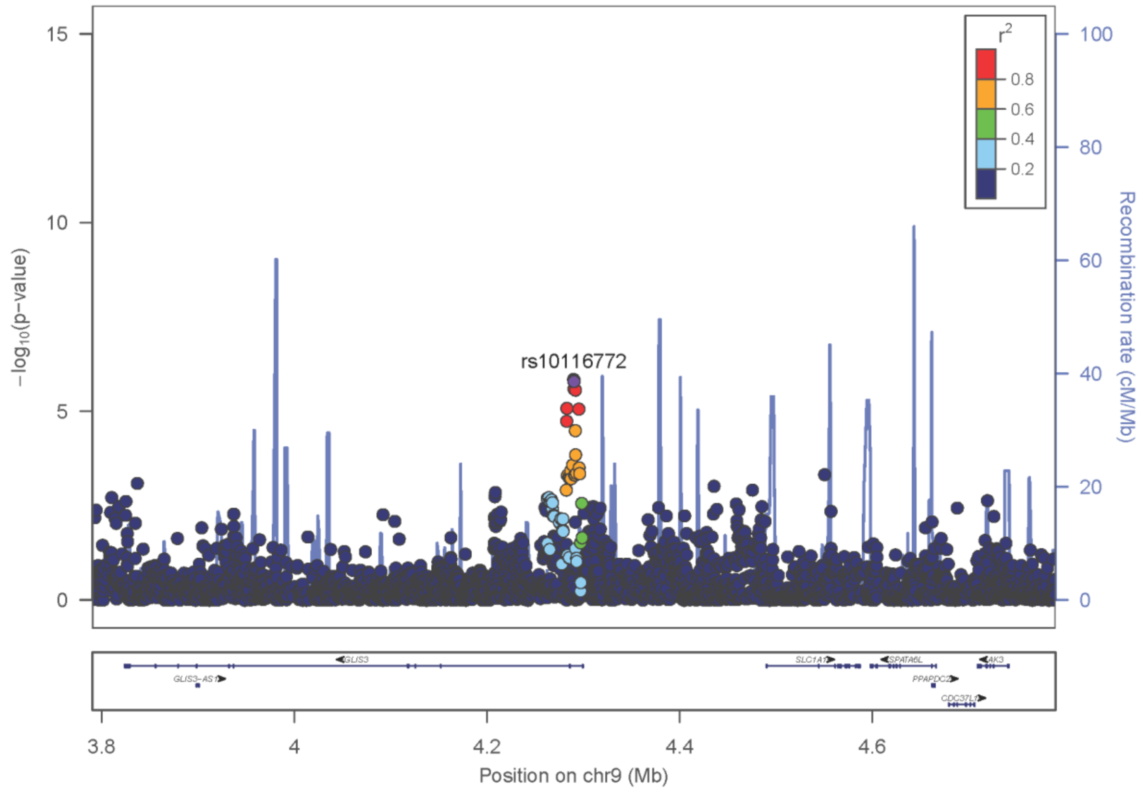

Figure S6. Imputed region of association around the index variant with osteoarthritis. The x axis shows the genomic interval, the left y axis shows the statistical significance of association as negative log10 of p values. rs10116772 was used as the reference SNP and its p value in the discovery set and post replication is denoted by the purple circle and diamond respectively. The linkage disequilibrium is presented as pairwise r^2 between the reference SNP and the other SNPs in the region with colours according to different bins (0-0.2, dark blue; 0.2-0.4, light blue; 0.4-0.6, green; 0.6-0.8, orange; 0.8-1.0, red). The plot was produced using LocusZoom.
